# Maternal Investment in the Swordtail Fish *Xiphophorus multilineatus*: Support for the Differential Allocation Hypothesis

**DOI:** 10.1371/journal.pone.0082723

**Published:** 2013-12-09

**Authors:** Oscar Rios-Cardenas, Jason Brewer, Molly R. Morris

**Affiliations:** 1 Red de Biología Evolutiva, Instituto de Ecología, A.C., Xalapa, Veracruz, Mexico; 2 Department of Biological Sciences, Ohio University, Athens, Ohio, United States of America; The Ohio State University, United States of America

## Abstract

The differential allocation hypothesis predicts that reproductive investment will be influenced by mate attractiveness, given a cost to reproduction and a tradeoff between current and future reproduction. We tested the differential allocation hypothesis in the swordtail fish *Xiphophorus multilineatus*, where males have genetically influenced (patroclinous inheritance) alternative mating tactics (ARTs) maintained by a tradeoff between being more attractive to females (mature later as larger courting males) and a higher probability of reaching sexual maturity (mature earlier as smaller sneaker males). Males in *X*. *multilineatus* do not provide parental care or other resources to the offspring. Allelic variation and copy number of the Mc4R gene on the *Y*-chromosome influences the size differences between males, however there is no variation in this gene on the *X*-chromosome. Therefore, to determine if mothers invested more in offspring of the larger courter males, we examined age to sexual maturity for daughters. We confirmed a tradeoff between number of offspring and female offspring’s age to sexual maturity, corroborating that there is a cost to reproduction. In addition, the ART of their fathers significantly influenced the age at which daughters reached sexual maturity, suggesting increased maternal investment to daughters of courter males. The differential allocation we detected was influenced by how long the wild-caught mother had been in the laboratory, as there was a brood order by father genotype (ART) interaction. These results suggest that females can adjust their reproductive investment strategy, and that differential allocation is context specific. We hypothesize that one of two aspects of laboratory conditions produced this shift: increased female condition due to higher quality diet, and/or assessment of future mating opportunities due to isolation from males.

## Introduction

According to life history theory, natural selection favors behaviors that maximize lifetime reproductive success based on several key tradeoffs. Such tradeoffs can be grouped in two main categories: between present and future reproduction (e.g. reproduction vs. growth, survival etc.), and within present reproduction, between quantity and quality of offspring (number of offspring vs. size, age at maturity, etc.) [1]. As explained by parental investment theory, the reason for these tradeoffs is that parental investment to current offspring reduces the survival and future reproductive success of the parents, and investment in many offspring reduces the quality of each offspring a parent can produce [2]. Therefore, parents facing decisions in terms of optimal resource allocation will often choose between current and future reproduction, basing the decision on the value of the current brood relative to the value of the parent’s expected future reproduction, and between quantity and quality, basing this decision on the value of many small offspring relative to few larger offspring [3]. In general terms, a solution to these parental investment decisions should be based on how choices influence the parent’s expected lifetime reproduction [3].

Provided that mothers can adjust the investment devoted to each offspring and that females have reliable cues predicting future environmental conditions, several factors have been proposed to determine facultative differential allocation by mothers [4,5]: 1) if larger females have more resources to allocate to reproduction, then they may produce larger broods and/or larger offspring [6-8]; 2) if females can assess their likelihood of future reproduction, then they should invest heavily in their last reproductive events; 3) finally, females are predicted to adjust their investment in offspring depending on the attractiveness of their mate, as long as there is a cost to reproduction, a tradeoff between current and future reproductive success and evidence that mating with high quality mates increases female fitness [9-12]. Empirical support for the differential allocation hypothesis continues to grow, including examples in a variety of taxa where investment is positively correlated with the attractiveness of the mate [13-15], as well as cases where this relationship is negative [16-18]. To determine factors that influence the direction of the relationship between investment and mate attractiveness, it will be essential to increase the number of empirical studies with clear measures of mate attractiveness [19]. In addition, to better understand selection for facultative adjustment of reproductive investment as well as its evolutionary consequences, we will need more empirical studies that can unite measures of mate preference with the benefits of the preferences [10,15,17,20]. 

We examined facultative maternal investment in the Northern swordtail *Xiphophorus multilineatus*, which is endemic to the Río Coy and its tributaries in the State of San Luis Potosi, Mexico. There is no paternal investment in this species, as males insert their intromittent organ, transfer a sperm packet, and then swim away [21]. Fishes of the genus *Xiphophorus* are lecithotrophic [22], which means that females provision the eggs in the form of yolk prior to internal fertilization; therefore, any facultative maternal investment allocations should also be made prior to fertilization. Two lecithotrophic species of fish responded to low food availability by producing smaller eggs [23]. In addition females store sperm [22], and so fertilization does not necessarily occur at the same time as mating. Therefore, *X. multilineatus* females could adjust investment in their eggs based on mating experiences before fertilization occurs.

This system is particularly suited for studying the direction of maternal investment in relation to male attractiveness as well as the evolutionary consequences of these investments for two reasons. First, it will be possible to predict the direction of allocation, as mating preferences for *X. multilineatus* have been studied extensively, with an overall strong preference for the larger courting males as compared to the smaller sneaker males [24,25]. Previous work has demonstrated that the strength of preference for the larger courter males increases with female size [24], which increases with female age [26], and that this variation in preference results in variation in male mating success in the field [27]. Second, detecting differential investment in a system with alternative reproductive tactics (ARTs) is of particular interest if maternal investment influences the traits that help maintain the differences between the male morphs. The larger courter males have a higher mating success (due to female choice [24], and male-male competition, [28]), while the smaller sneaker males increase their probability of surviving to sexual maturity by maturing earlier at smaller sizes [29]. Therefore, one important evolutionary consequence of facultative maternal investment could be that the size and or growth differences between the ARTs are influenced by differential allocation based on mate attractiveness. 

The differential allocation hypothesis [9,30] suggests that reproductive investment will be influenced by mate attractiveness in *X. multilineatus*. This hypothesis is based on the assumptions of there being a cost to reproduction and a tradeoff between current and future reproductive success [30]. Therefore, we first determined if there was a cost of reproduction by looking for a tradeoff between number of offspring and one of two potential measures of offspring quality (size or age to sexual maturity). Second, we tested the differential allocation hypothesis by determining if females invested differently in the daughters of the more attractive courter males as compared to daughters of the sneaker males. The courter and sneaker males are genetically influenced ARTs with patroclinous inheritance, resulting in the male ARTs breeding true [32,33]. Therefore, to distinguish between the influences of genetic variation on the *Y*-chromosome associated with the ARTs and maternal investment, we assumed that investment in the eggs would occur before females could determine if they were investing in males or females and assessed investment in daughters, as the Mc4R gene does not vary on the *X*-chromosome [33]. And finally, we determined if individual females changed their investment strategies by comparing the first brood (provisioned in the wild) to future broods provisioned in isolation from males in the laboratory.

## Materials and Methods

### Ethics statement

The “Secretaria de Agricultura, Ganadería, Desarrollo Rural, Pesca y Alimentación (SAGARPA)” issued the necessary permission to do this research (permit number DGOPA/00132/1001107.0032). All experiments comply with the laws of the United States and the Animal Care Guidelines of Ohio University and was approved by the Institutional Animal Care and Use Committee (IACUC Use no. L01-01).

In January 2007, using seines and minnow traps, we caught gravid *X. multilineatus* females from the Oxitipa river (N 21° 41.868, W 99° 0.504) and transported them to our facilities at Ohio University. We measured the female’s standard length (SL), placed them in individual, visually isolated 18.9 l tanks, and fed them to satiation daily with TetraMin flakes. Female’s tanks were checked daily for fry. Once a brood was dropped (up to eight individuals within minutes) we moved the female to a new tank. If brood size was larger than four fry, we split the brood into two tanks within the first 2 months of their life. Females can continue to drop broods in isolation using stored sperm. Therefore, we followed each female and the offspring from all of their broods for the next 9 months. During this time we compiled a reproductive history for the females and their offspring, recording brood size, total number of broods each female dropped, number of tank mates (number of fish in tank where individual was raised to sexual maturity), and mother’s standard length at time each brood was dropped.

When fry reached sexual maturity, brood spot development for females and gonopodial formation for males [31], we recorded the date and their SL (age and size at sexual maturity) and we determined sex ratio for each brood. Daughters were removed from their home tanks and placed in communal tanks while the males were removed and individually isolated until their genotype could be definitively assigned based on size at sexual maturity and pigment patterns [32]. As the alternative reproductive tactics in this species have patroclinous inheritance [26,33]) with the smallest size class (sneakers) differing from the three larger size classes (courters) in the pigment pattern vertical bars [34,35], body depth, dorsal fin size, and length of the sword [26,32], we could use the genotype of the sons a female produced to determine the genotype (hereafter ART) of the male(s) that sired the offspring for each female. The ART of the father for each offspring was scored as one of three possible states: sneaker as the sire (all brothers sneakers), both sneaker and courter sires possible (both sneaker and courter brothers), and courter as the only sire (all brothers courters). 

We used the F1 daughters to test maternal investment predictions, as females are all homozygous for the small size alleles on the *X*-chromosome [33], and therefore more of the variation among daughters in age and size at sexual maturity should be due to environmental factors such as maternal investment. First we determined if there was a cost to reproduction by confirming that there was a tradeoff between quantity and quality of the offspring. For this we analyzed the relation between number of offspring (a quantity estimator), and their size and age at sexual maturity (two quality estimators). Second, we determined the relationship between mother size (estimated by their SL) and brood size (total number of fry dropped). We restricted this analysis to the females that dropped fry within the first 30 days in the lab to avoid the potential effects of adjustments in investment that females may make once they were under our experimental laboratory conditions. Based on a gestation period of 30 days [22], females would have already provisioned these broods prior to being captured. Third, we examined female allocation to offspring after they had been physically and visually isolated from other fish in the laboratory, which constrained them to the use of stored sperm. Thus, we used brood order as an estimator of decreased expected future reproductive success (sperm should be either depleted or degraded with time). Finally, we also included the father’s ART in our analyses to test the differential allocation hypothesis. To avoid pseudo replication and the lack of independence of data coming from the same brood or the same mother, for these analyses we obtained average values for each brood and randomly selected the values of only one brood from each individual mother that had multiple broods. The analyses included a weighting scheme that considered the number of fry from where we obtained the per-brood averages.

We examined factors influencing variation in brood size and age at maturity with two Generalized Linear Models (GzLM) that included the independent variables (brood order and father’s ART), the weighting scheme and its respective covariables (see below for the specific covariables used in each case). For the analysis of brood size we used a GzLM with a Poisson distribution, a log link function, and mother SL as a covariable. For the analysis of age at maturity we used a GzLM with a normal distribution, an identity link function, and brood size, mother SL, number of tank mates (see above), and number of sisters and brothers (not all sibling were raised in the same individual tank) as covariables. We started the analyses with a model that included all predictor variables (a maximal model that included the explanatory variables and all covariates) and then found the minimal adequate model. Using a model simplification based on the Akaike’s Information Criterion (AIC), we long-hand assessed the significance of the increase in deviance that results when a given term was removed from the current model. We report the minimal adequate models based on decreasing the AIC [36]

## Results

The reproductive data of the wild-caught females (mothers in the current study) have been reported elsewhere [27]. Thirty-seven females produced one brood, 27 two, 17 three, 10 four and one female produced five broods, for a total of 92 broods and 307 fry. The average size of a brood was 3.34 fry, with 8 being the maximum number of fry per brood. The average number of days between broods (interbrood interval) was 59.95 days (minimum 40 days, maximum 130 days). Among all broods, sex ratio ranged from 0.42-0.54 (males:females) with an average of 0.47 [27]. Eighteen females were mated by only courter males, 9 females by only sneaker males and 5 females had fry sired by both courters and sneaker males. We could not determine the ART of the fathers of the fry produced by four of the females as no sons were produced or survived to be classified; thus we only include these broods in the analysis that involved the effect of mother size on brood size, and then removed them from subsequent analyses.

 Two hundred and fifty one fry were raised to sexual maturity, and 133 of these were daughters. Brood size was not significantly related to size at maturity of daughters (Weighted linear regression: *R* = 0.177, *F*_1, 28_ = 0.869, α’ = 0.025 after Bonferroni correction, *P* = 0.36) but it was significantly related to age at maturity (Weighted Linear regression: *R* = 0.573, *F*_1, 28_ = 13.217, α’ = 0.025 after Bonferroni correction, *P* < 0.01), with age to maturity increasing with brood size. Therefore, given that we detected a relationship between brood size and age at maturity, we further examined the factors influencing these two traits.

For the first broods females produced, there was a significant relationship between mother’s standard length and brood size (Linear regression: *R* = 0.753, *F*_1, 15_ = 18.294, α’ = 0.017 after Bonferroni correction, *P* < 0.001) with brood size increasing with female size. We did not detect a significant relationship between mother size and the size of their daughters (Linear regression: *R* = 0.399, *F*_1, 16_ = 2.836, α’ = 0.017 after Bonferroni correction, *P* = 0.11) or age at maturity of their daughters (Linear regression: *R* = 0.426, *F*_1, 16_ = 3.328, α’ = 0.017 after Bonferroni correction, *P* = 0.09).

**Figure 1:**
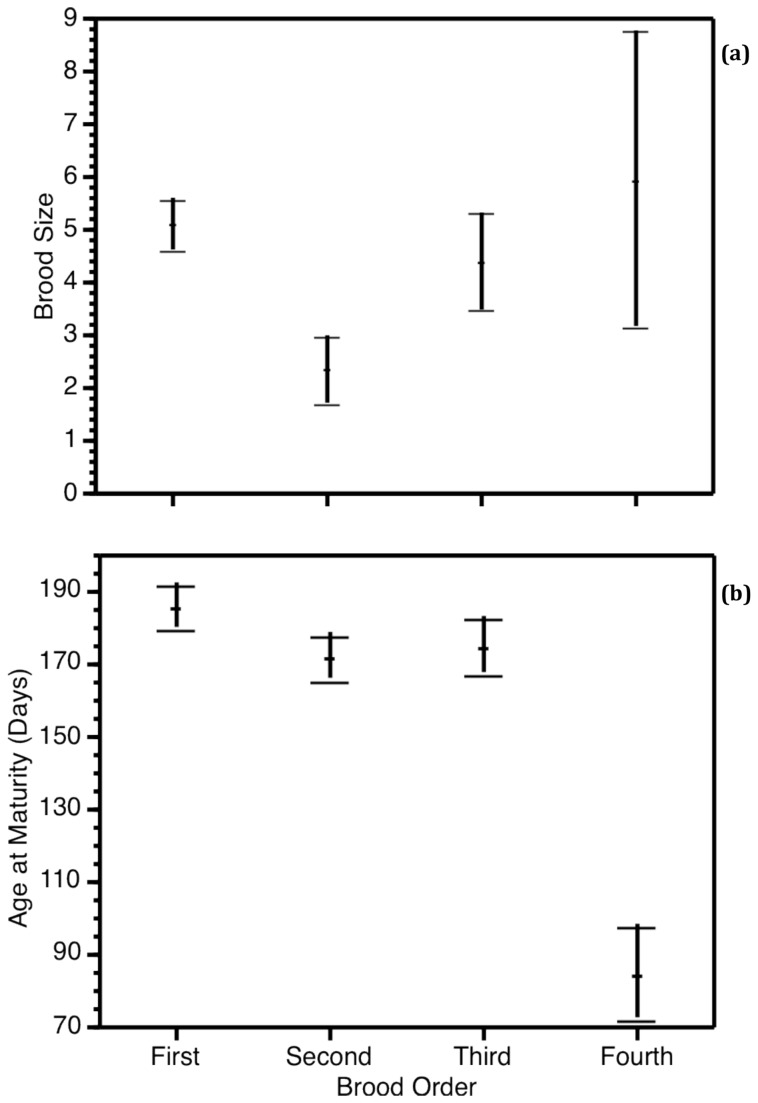
Female allocation to offspring based on decreased expected future reproductive success. The effect of brood order on the number of fry dropped (a) and age at maturity (b) (means ± standard errors).

**Figure 2:**
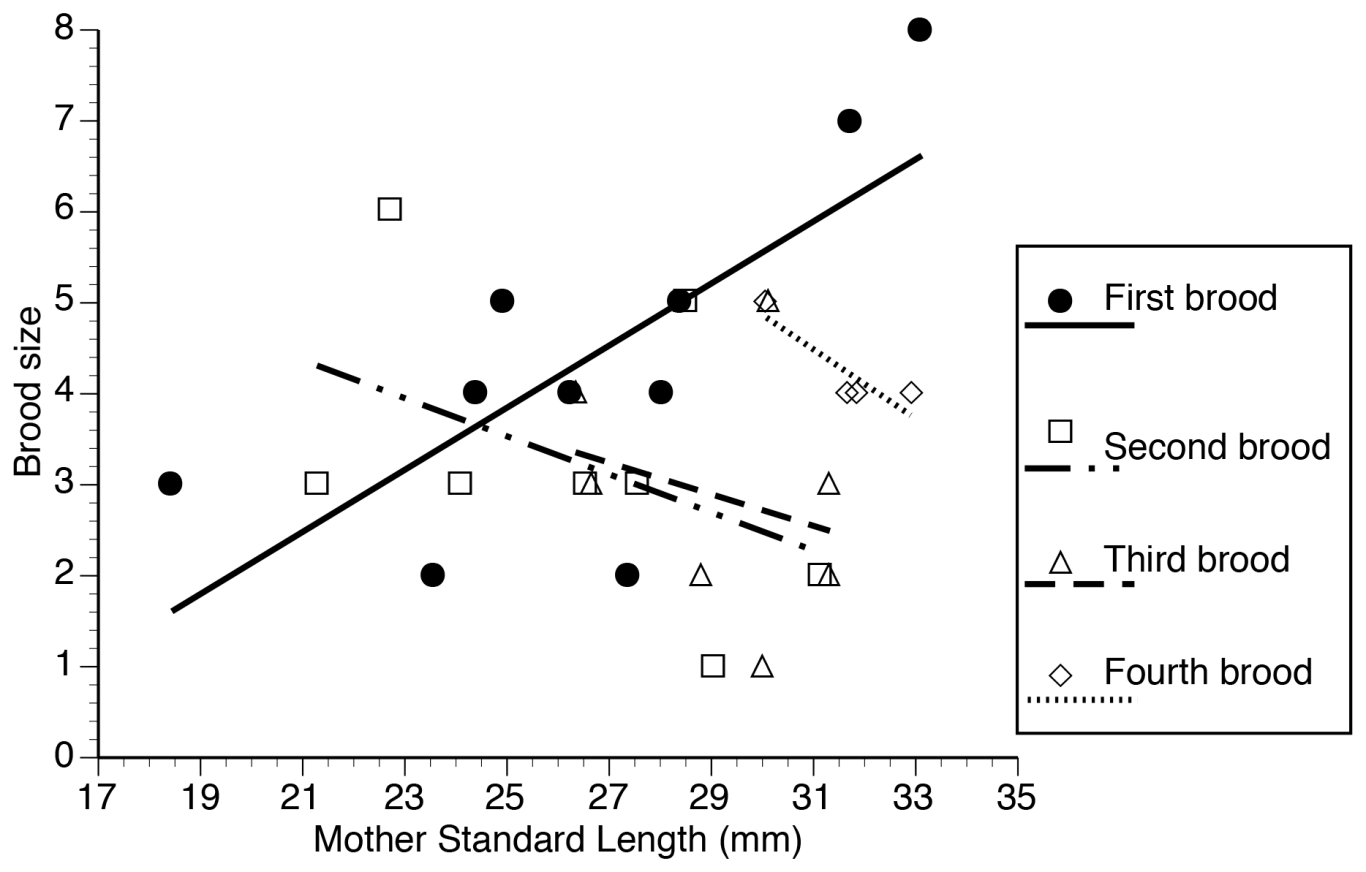
Relationship between mother size and number of fry dropped (brood size) by females that had four broods. Lines represent predicted values based on linear regression.

 Brood order significantly affected brood size (table 1); specifically, brood size decreased for the second brood, but subsequent broods were not significantly smaller than first broods (Bonferroni multiple comparisons *P* = 0.004; Figure 1a). We found a significant interaction between brood order and mother standard length affecting brood size (table 1). An examination of the graphic representation of this interaction confirms that for the first brood, brood size increases with mother size (see above), but that such a relationship did not occur in subsequent broods (Figure 2). We compared the slope of the relation between mother standard length and brood size for the first brood with the slope for the second, third and fourth broods (pooled together). There was a significant difference in the slopes (Heterogeneity of slopes test: *F*_1,25_ = 6.09, *P* = 0.02). The effect of brood order on brood size remained significant even after pooling broods with a similar slope in the analysis (data not shown).

**Table 1:** Test of the model effects for brood size as response variable.

Source	Likelihood Ratio Chi-Square	df	*P*
(Intercept)	10.01	1	0.002
Brood Order	13.916	3	0.003
Father’s ART	1.983	2	0.371
Mother Standard Length (Covariable)	3.497	1	0.061
Brood Order * Father’s ART	6.754	3	0.08
Brood Order * Mother Standard Length	15.766	3	0.001

df = degrees of freedom

**Figure 3:**
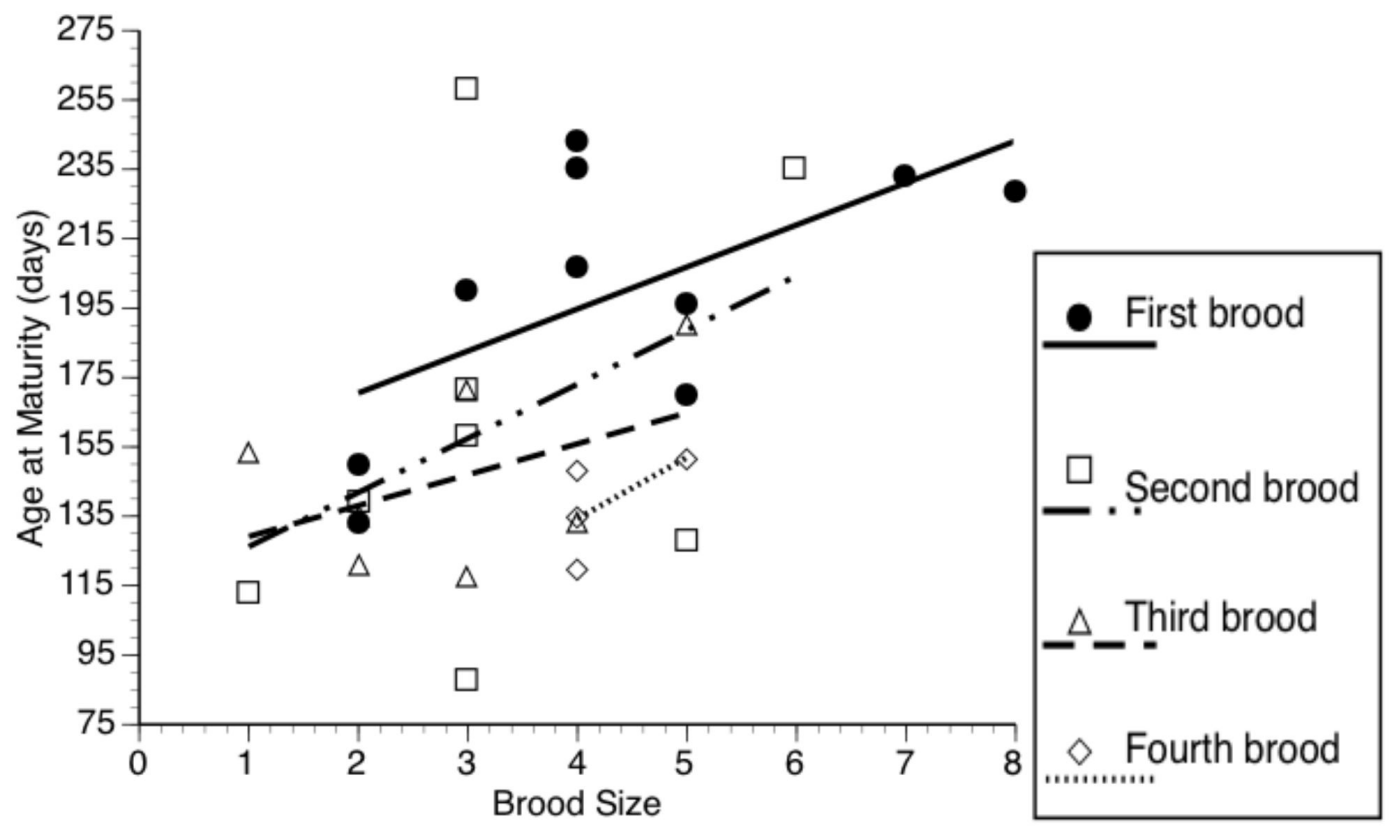
Relationship between numbers of fry dropped (brood size) and age at maturity for the different broods. Lines represent predicted values based on linear regression.

For the analysis of age at maturity, the only covariables included in the final model and thereby controlled for, were brood size and number of tank mates, as there was a statistically significant relationship between these covariables and age at maturity (table 2); mother SL, number of sisters and number of brothers did not have a significant effect and did not increase the information criteria, thus these covariables were removed from the final model. Brood order had a significant effect on age at maturity (table 2); particularly, daughters from the last brood matured at an early age (Bonferroni multiple comparisons *P* < 0.001; Figure 1b). We also found a significant interaction between brood order and brood size (table 2). An examination of the graphic representation of this interaction confirmed that age at maturity increases with brood size (see above), and that even though the slope for this relationship is different for the different broods, the general direction of the relationship is similar among the broods (Figure 3).

**Table 2:** Test of the model effects for age at maturity as dependent variable.

Source	Likelihood Ratio Chi-Square	df	*P*
(Intercept)	15.394	1	< 0.001
Brood Order	28.608	3	< 0.001
Father’s ART	9.484	2	0.009
Brood Size (Covariable)	5.26	1	0.022
Tank Mates (Covariable)	23.258	1	< 0.001
Brood Order * Father’s ART	24.706	3	< 0.001
Brood Order * Brood Size	27.3	3	< 0.001

df = degrees of freedom

**Figure 4:**
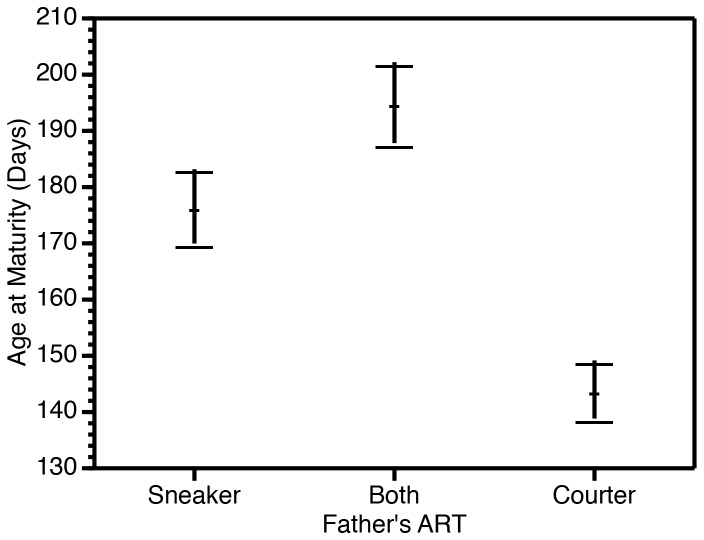
Effect of father’s alternative reproductive tactic (ART) on age at maturity (means ± standard errors).

 Father’s ART had no significant effect on brood size (table 1). However, the father’s ART had a significant effect on age at maturity (table 2), as daughters sired by courters reached sexual maturity sooner (Bonferroni multiple comparisons *P* < 0.001; Figure 4). As the genetic differences between courters and sneakers are only passed on to the sons, our results suggest that females mated to courters invested more in individual offspring. We also found a significant interaction between brood order and father’s ART (table 2). An examination of the graphic representation of this interaction (not shown) revealed that the decrease in age at maturity for the daughters of females mated with courters was not detected in the first brood, but only in later broods. 

## Discussion

The differential allocation hypothesis as originally suggested by Burley [9] and more broadly defined by Sheldon [30] suggests that parents tradeoff current and future reproduction, and that the attractiveness of the mate will influence this tradeoff. Our study revealed a shift from investing in offspring number (larger brood sizes) to offspring quality (daughters reached sexual maturity sooner) depending on whether females provisioned their offspring in the wild (first broods) as compared to the context of the laboratory (subsequent broods). Brood size was only correlated with female size in the first brood, and age to sexual maturity was significantly less (higher quality offspring) in later broods. In addition, the shift to investing in higher quality female offspring was the most pronounced for females that had mated with courters. The larger courter males in *X. multilineatus* are on average strongly preferred by the females of this species [24], and do not pass on the genes influencing ARTs to their daughters. Therefore, the increase in quality of daughters of females that mated to courter males supports the differential allocation hypothesis [9]. Below, we consider potential hypotheses for factors that influenced the shift in allocation strategies between offspring provisioned in the wild (first brood) and the laboratory, including a tradeoff between current and future reproduction.

One possible explanation for the shift in investment between the wild and laboratory contexts is food quality and quantity, as both are likely to be lower in the wild than the laboratory. It is interesting to note, however, that even though feeding remained constant while females were housed in the laboratory, the longer females were in the laboratory producing offspring from stored sperm, the more they invested in individual offspring. In addition, brood size was not significantly smaller in the last two broods (produced from stored sperm) as compared to the first brood (females more likely to have recently mated). This suggests that females were not sperm limited, and that females were not reducing offspring number to increase offspring quality. Therefore, while differences in food quality and quantity can explain the ability to increase offspring quality in lab provisioned broods as a whole, an additional difference between wild and laboratory is needed to explain the increased investment in offspring quality with subsequent laboratory provisioned broods.

 The shift to investing more in individual offspring could represent females trading off future reproduction for current reproduction. With each subsequent brood produced in the laboratory, the time since last associating and or mating with a male increased. Therefore, females may have perceived that their future reproductive opportunities were limited due to their isolation from males in the laboratory. State-based models of female reproductive investment suggest that perceived potential for future reproduction should influence patterns of reproductive investment [10]. Similar context dependent differential allocation patterns have been detected previously, but not within the same study. For example Cunningham and Russell [37] found that young female mallards increased their egg volume when mated with highly attractive males but Bluhm and Gowaty [38] demonstrated that older females increase their egg volume when mated with less attractive males. 

In summary, we suggest that maternal investment adjustments in brood size in *X. multilineatus* are based on female body size, while adjustments to offspring quality are influenced by interactions between food quality, probability of future reproduction (mating), as well as the quality of male mated. Our hypothesis would predict extensive variation in maternal investment across both space and time, a prediction that is supported by findings in another species of swordtail, *X. birchmanni* [39]. Larger, older females produced larger broods and larger offspring than younger females, but the relationship between female size and offspring size was not detected across all the sites examined [39]. A previous study of the breeding cycles of wild caught *X. multilineatus* also found a significant relationship between female size and brood size, and yet noted that brood size varied extensively by season [40].

A tradeoff between number and quality of offspring has been documented across many different taxa [1], as well as for other live-bearing fish [41]. The clear tradeoff between number of offspring and age to sexual maturity of daughters we detected supported our interpretation of this trait as a measure of maternal investment in offspring quality. Age to sexual maturity is an important life-history trait for females, as the longer it takes females to reach sexual maturity, the less likely they are to survive to reproduce.

Our results suggest either some advantage to producing higher quality daughters of courter sires, or an inability to invest differentially in male and female offspring, such that female offspring of courter sires are gaining an advantage simply due to the benefits of increased investment in their brothers. Due to the confounding factors of patroclinous inheritance of age at sexual maturity and thus size for sons [26,33], we only considered daughters in our analysis of mother’s investment. However, while the haplotype of the *Y-*chromosome has a large influence on age/size at sexual maturity of sons, there is still extensive variation around the means for each size class [32]; thus, maternal investment could influence sons as well as daughters, just to a lesser degree. In particular, if the influence of maternal allocation is the same for male as compared to female embryos it could explain in part the faster growth rate of courter males as compared to sneaker males in this species [29].

It has been argued that tests of the differential allocation hypothesis should be experimental, either manipulating male attractiveness or assigning mates experimentally, so as to control for the possibility that investing more in the offspring of attractive mates is due solely to a relationship between female quality and mate quality [30]. In our system larger females are more likely to mate with courter males [27], and therefore a relationship between female quality and the preferred courter males is expected. However, by controlling statistically for the influence of female size on the relationship between father’s ART and daughter’s age at sexual maturity (i.e., offspring quality), we were able to determine that the relationship between daughter’s age at sexual maturity and father’s ART was not due primarily to mother’s size.

The selection against intermediate expression of reproductive traits, or disruptive selection, is expected to lead to the evolution of ARTs [42]. Therefore, the degree to which differential maternal investment increases the disruptive selection between preferred and non-preferred males could suggest a role for maternal investment in the evolution of ARTs. In those systems where the inheritance of condition results in the alteration of tactics across generations, negative maternal and paternal effects can help maintain the ARTs [42]. The ARTs in *X. multilineatus* are genetically influenced by genes on the *Y*-chromosome [32], and are maintained at least in part by a tradeoff between being more attractive (courters males) and reaching sexual maturity sooner (sneaker males). Therefore, mothers that provide the sons of courters with more resources to reach sexual maturity sooner could increase the overall reproductive success of their courter sons as compared to their sneaker sons. The decision to withhold resources or increase maternal investment may involve an interaction between not only mate quality and a female’s reproductive life history, but also the availability of attractive males within a population, or the degree of reproductive skew [10]. For example, previous authors have suggested that reproductive compensation is more likely to occur in situations where females are not allowed to choose their mates [10,30,43]. If sneaker males are more common, or the only existing type of male, which is the case in two species of swordtails (e.g. *X. pygmaeus* and *X. continens*), their success at circumventing female mating preferences could act to decrease maternal investment in preferred males (differential allocation), driving females to use a strategy of reproductive compensation. Comparisons across populations of *X. multilineatus* with different frequencies of courter/sneaker males, as well as with *X. pygmaeus* and *X. continens* will provide valuable tests of these hypotheses.
